# Interleukin-37 Serum Levels in Oral Squamous Cell Carcinoma 

**DOI:** 10.30476/dentjods.2025.105421.2595

**Published:** 2026-09-01

**Authors:** Hamid Ghaderi, Mohammad Javad Fattahi, Hossain Shirmardi Dezaki, Mahyar Malekzadeh, Bijan Khademi, Abbas Ghaderi, Mohammad Reza Haghshenas

**Affiliations:** 1 Violet Vines Marshman Centre for Rural Health Research, La Trobe University, Bendigo, Australia.; 2 Shiraz Institute for Cancer Research, School of Medicine, Shiraz University of Medical Sciences, Shiraz, Iran.; 3 Otolaryngology Research Center, Dept. of Otolaryngology, Shiraz University of Medical Sciences, Shiraz, Iran.

**Keywords:** Oral Squamous Cell Carcinoma, Interleukin 37 Protein, Enzyme-Linked Immunosorbent Assay

## Abstract

**Background:**

Squamous cell carcinoma (SCC) is the most common cancer of the maxillofacial area, known for its high mortality and morbidity rates. Increasing evidence highlights the critical role of chronic inflammation in human malignancies by modulating inflammatory cells and cytokine synthesis. IL-37 is recognized as a cytokine that may play a dual role in cancer.

**Purpose:**

This study sought to investigate IL-37 serum concentrations in individuals with oral SCC, specifically SCC of the tongue.

**Materials and Method:**

This case-control study investigated IL-37 serum levels and their association with the patients' clinicopathological characteristics in 65 cases of oral SCC compared to 65 healthy controls using the ELISA technique. The study further assessed the diagnostic significance of IL-37 serum concentrations using receiver operating characteristic (ROC) curve analysis.

**Results:**

The results indicated that IL-37 serum concentrations were markedly higher in patients than in the control group, although no association with the patients' clinicopathological characteristics was found. The best cut-off point for the serum IL-37 level was 61.95 pg/ml, with a sensitivity of 92.30% and specificity of 87.70%. The ROC curve area was 0.90.

**Conclusion:**

The significant increase of serum IL-37 levels in oral SCC patients underscores its involvement in the disease's pathology and suggests its potential use in early detection and screening. Future studies should aim to elucidate the interplay of this cytokine with the tumor microenvironment (TME) and explore its possible diagnostic and therapeutic applications in cancer.

## Introduction

Head and neck malignancies contribute significantly to the burden of disease worldwide with 90% of lesions identified as squamous cell carcinoma (SCC) [ 1 ]. These highly invasive malignancies can originate from the lips, tongue, the floor of the mouth, tonsils, and salivary glands [ 2 - 3 ]. SCC is marked by its aggressive behavior, with a significant proportion of cases diagnosed at advanced stages involving multiple lymph nodes, leading to a low five-year survival rate [ 4 - 5 ]. The absence of tumor-specific biomarkers adds further to these challenges, hindering early detection and limiting options for targeted therapies [ 6 ]. Tumor biomarkers can be determined at metabolomics, proteomics, and genomics levels and can play a part in risk assessment, screening, diagnosis, prognosis, and monitoring cancer therapy response. Consequently, the identification and development of SCC-specific tumor biomarkers could significantly improve patient survival outcomes [ 7 - 8 ]. Chronic inflammation has proven to play a particular role in human malignancies, with cytokines playing a crucial role in shaping this inflammatory milieu. Similarly, a transition to the inflammatory milieu as well as overexpression of several chemokine-cytokine receptors was observed in SCC patients [ 9 - 11 ]. Increased serum concentrations of pro-inflammatory cytokines, such as interleukin (IL)-1, IL-6, IL-8, and tumor necrosis factor alpha (TNF-α), frequently linked to cancer advancement and unfavorable clinical outcomes [ 12 - 14 ]. Notably, IL-1family has attracted considerable interest, mediating both pro-inflammatory and anti-inflammatory responses. Among its members, IL-37, a newly identified cytokine, stands out from others like IL-1α, IL-1β, and Il-18 by exerting unique anti-inflammatory effects [ 15 - 16 ]. Recognized for its unique properties, IL-37 demonstrates significant anti-inflammatory and immune-inhibitory role across a variety of autoimmune disorders [ 17 - 20 ]. The role of IL-37 in human malignancies is not well established, however, the anti-inflammatory properties of IL37 suggest a potential tumorigenesis/tumor suppressive role for this cytokine [ 21 ]. While previous studies have highlighted IL-37’s protective effects in various cancers including cervical, lung, and colon cancers, its precise role and signaling pathways in oral SCC are remained unclear [ 22 - 24 ]. A previous report investigated IL-37 expression in individuals with SCC of oral cavity, comparing it with oral leukoplakia (OLK) and normal controls using immunohistochemistry analysis. Results indicated an increasing trend in the expression of IL-37from normal oral mucosa, to OLK and to SCC. Conversely, IL-37 expression showed an inverse relationship with lymph node metastasis in oral SCC, with IL-37 levels being lower in cases exhibiting lymph node metastasis compared to those without [ 25 ]. Serum and tissue samples are among the most accessible and reliable sources for investigating potential biomarkers for human malignancies [ 26 - 27 ]. In patients with oral SCC, IL-37 serum levels were notably lower compared to those in individuals with OLK and in normal control groups. Additionally, an elevated IL-18 to IL-37 ratio, defined by increased IL-18 levels and decreased IL-37 levels, was associated with lymph node metastasis and advanced tumor stages [ 28 ]. Similarly, reduced IL-37 levels have been observed in renal cell carcinoma, colon cancer, multiple myeloma (MM), and hepatocellular carcinoma (HCC) [ 29 - 32 ]. Conversely, several malignancies, including epithelial ovarian cancer (EOC) and gastric cancer, have shown increased serum expression of IL-37 [ 33 - 34 ]. Moreover, our previous studies demonstrated elevated IL-37 serum levels in patients with primary brain tumors, endometrial cancer, and bladder transitional cell carcinoma [ 35 - 37 ]. Research on the role of IL-37 in SCC has been limited. Therefore, the objective of this study was to examine IL-37 levels in the serum of patients with oral SCC, particularly those originating from the tongue (SCCOT), and analyze its correlation with patients’ clinicopathological parameters. 

## Materials and Method

The current case-control study involved 65 newly diagnosed oral SCC patients (mean age ±SD: 63.75±15.44, ranging from 27 to 88 years) who were admitted to Khalili Hospital, affiliated with Shiraz University of Medical Sciences and 65 healthy individuals (mean age ±SD 60.72±13.50, with ages ranging from 30 to 89 years) as the control group. The diagnosis was confirmed by both a surgeon and a pathologist at Khalili Hospital. Subsequently, blood samples were transferred to the Shiraz Institute for Cancer Research for further investigation. Patients with previous history of cancer, previous chemo/radiation therapy, manifestation of infectious diseases within the past thirty days, and history of autoimmune disease were excluded from the study. The control group included healthy donors without a family history of cancer, autoimmune diseases, or recent infection. The study was authorized by the Shiraz University of Medical Sciences' Ethics Committee (IR. sums. med.rec.1398.302) and informed consent was obtained from all participants.

Blood sample of 5 mL was drawn from each volunteer, and serum was isolated through centrifugation at 2500 RPM for 10 minutes at a temperature of 37°C. The isolated serum samples were then frozen and stored at -70°C for later cytokine analysis. Serum levels of IL37 were then quantified with a commercial human IL-37 ELISA kit (Shanghaicrystal Day Biotech CO., LTD), following the manufacturer’s protocol. The assay range for this ELISA kit was from 7pg/ml up to 400 pg/ml, with a sensitivity of 4.5pg/ml. 

All statistical analyses were performed using the SPSS software package (version 11.5; SPSS Inc., Chicago, IL, USA). To examine and compare findings between groups, non-parametric tests, specifically the Mann–Whitney U and Kruskal–Wallis H tests, were employed. The correlation between IL-37 and tumor size and age was determined using non-parametric Spearman rank correlation. Values are expressed as mean ± SEM, and *P* values less than 0.05 is considered as statistically significant. 

## Results

The study included a total of 130 participants, consisting of 65 patients with oral SCC and 65 healthy individuals as controls. The tumor site in the majority of patients with SCC was tongue (63 patients, 96.9%). Among the patient group, 38 individuals had well-differentiated tumors, representing the majority of patients (58.46%). The highest number of cases (23 patients, 35.38%) were diagnosed at stage III. Of the patients, 22 showed lymph node involvement, 14 had lymphovascular invasion, and 18 presented with peripheral invasion. 

As indicated in Table 1, patients demonstrated significantly higher mean IL-37 serum levels (107.61±7.45 pg/ml) compared to a control group (42.93±4.33 pg/ml, *p*< 0.05). Nevertheless, IL-37 serum levels showed no statistically significant variation between genders in either the patient or control groups (*p*= 0.93 and *p*= 0.50, respectively). 

**Table 1:** Comparison of IL-37 levels in patients with oral squamous cell carcinoma (SCC) and control group

Group	N	Mean±Std. Error	Median	Maximum	Minimum	*p* Value
Patient	65	107.61±7.45	92.70	310.82	6.09	0.001
Control	65	42.93±4.33	31.42	229.30	5.74	

IL-37 serum levels were analyzed across different tumor stages of oral SCC. The mean IL-37 serum level was 103.30±20.55 in patients with stage I, 135.26± 24.18 in stage II, 94.95±11.73 in stage III, and 129.00 ± 23.12 in stage IV. These differences were not statistically significant (*p*= 0.18). Additionally, IL-37 serum levels varied across different tumor grades of oral SCC as 113.88±11.47 in well-differentiated tumors, 105.19±10 in moderately differentiated tumors, and 68.61±33.96 in poorly differentiated tumors. Nevertheless, these variations were not found to be significant (*p*= 0.74). In addition, study found no significant differences between the different statuses of T stage, lymph node involvement, lymphovascular invasion, and peripheral invasion. Similarly, no significant association was identified between tumor size and IL-37 serum levels in this study. Data are summarized in Table 2. 

**Table 2:** Association between IL-37 serum levels and clinicopathological characteristics in patients with oral squamous cell carcinoma (SCC)

Parameters	Categories	N	IL-37 serum levels (pg/ml)			*p* Value
			Mean±Std error	Min	Max	
TNM Stage	Stage I	11	103.30 ± 20.55	64.10	303.98	0.18
	Stage II	9	135.26 ± 24.18	69.95	273.14	
	Stage III	23	94.95 ± 11.73	6.09	307.75	
	Stage IV	10	129.00 ± 23.12	67.47	310.82	
	Early stage	20	117.±15.69	64.01	330.98	0.97
	Late stage	33	105.2±1099	6.09	310.82	
Histological grade	Well Differentiated	38	113.88 ± 11.47	6.09	310.82	0.74
	Moderately Differentiated	18	105.19 ± 10.19	55.57	237.64	
	Poorly Differentiated	2	68.61 ± 33.96	34.65	102.57	
T Stage	T1	17	100.32 ± 13.73	63.01	303.98	0.12
	T2	26	118.00 ± 12.98	6.09	307.75	
	T3	19	102.79 ± 13.45	34.65	310.82	
Lymph node involvement	Seen	22	109.25±16.11	6.09	310.82	0.99
	Not seen	31	106.18±10.47	47.83	303.98	
Lymphovascular involvement	Seen	14	94.06±9.81	6.09	160.09	0.88
	Not seen	39	114.26±11.33	45.26	310.82	
Peripheral invasion	Seen	18	108.84±12.90	63.01	310.82	0.69
	Not seen	35	113.29±11.55	6.09	301.75	

**Figure 1:**
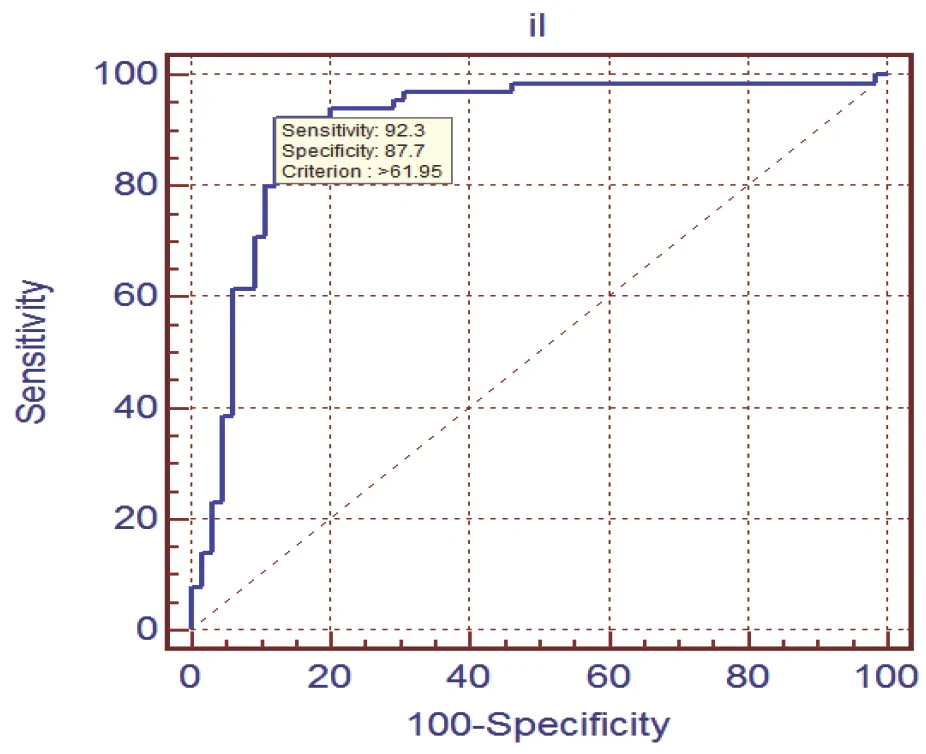
Receiver operating characteristic (ROC) curve for assessing specificity and sensitivity of IL-37 serum levels in patients with oral squamous cell carcinoma (SCC). The best cut-off point was 61.95 pg/ml, with a sensitivity of 92.3% and specificity of 87.7%. The lower level of the ROC curve for IL-37 serum levels is 0.90, indicating the high accuracy of IL-37 as an indicator for oral SCC

The study further assessed the diagnostic value of serum IL-37 using ROC analysis. As shown in Figure 1, the study identified 61.95 pg/ml as the optimal cut-off point for IL-37 serum levels, providing sensitivity and specificity of 92.3% and 87.7%, respectively. The area under the ROC curve for IL-37 levels stands at 0.90, underscoring the strong accuracy of IL-37 as a biomarker for oral SCC. 

## Discussion

This case-control study observed significantly higher IL-37 serum concentrations in oral SCC patients compared to healthy controls. However, IL-37 serum levels showed no correlation with the clinicopathological characteristics of the disease. IL-37’s role in human malignancies, including head and neck SCC, has been a subject of growing interest due to its dual function in modulating inflammation and potentially influencing tumorigenesis. IL-37 exhibits a complex and multifaceted role across various human malignancies, influenced by the specific context and tumor environment [ 21 ]. 

A 2015 study demonstrated that IL-37 suppresses the growth and invasion of cervical cancer cells by modulating the signal transducer and activator of transcription 3 (STAT3) signaling pathway [ 22 ]. Similarly, a study in 2014 indicated reduced IL-37 expression in primary HCC patients, showing a marked inverse association between tumor size and IL-37 levels, suggesting a potential tumor-suppressive role in HCC [ 38 ]. The study by Ge *et al* . [ 39 ] examined the role of IL-37 in non-small cell lung cancer, finding a significant reduction in IL-37 mRNA levels and protein expression, along with a negative correlation between IL-37 expression and overall patient survival. This evidence further reinforces IL-37’s inhibitory role in cancer progression. IL-37’s dual function in cancer biology may stem from its complex interplay with components of the tumor microenvironment (TME) [ 21 ]. IL-37’ overexpression in different cell types, such as macrophages and epithelial cells, align with its ability to modulate both innate and adaptive immune responses, indicating that IL-37 may either suppress or facilitate tumor growth depending on the particular cytokine environment and cellular context [ 21 ]. While the exact role of IL-37 in oral SCC remains unclear, its anti-inflammatory properties may contribute to tumor suppression. Lin *et al* . [ 25 ], using immunohistochemistry (IHC), investigated the expression of IL-37 expression in oral SCC and observed a significantly elevated IL-37 tissue expression in oral SCC than OLK and healthy oral mucosa. The study also observed a negative correlation between lymph node involvement and IL37 expression, suggesting that higher IL-37 levels might be associated with less aggressive tumor phenotypes [ 25 ]. Our study further supports these findings, as oral SCC patients exhibited significantly higher serum levels of IL-37 compared to healthy controls. This elevation in serum IL-37 could be indicative of its role in the immune response to tumor progression. Contrary to our findings, a study by Ding *et al* . [ 28 ] observed significantly higher IL-37 serum levels in healthy controls compared to those with OLK and in oral SCC. While differences in IL-37 expression levels may be attributed to variations in clinical stages, histological grades, and the diversity of tumor microenvironments among study patients, these discrepancies warrant further investigation employing a larger sample size to clarify the precise role of this cytokine in oral SCC. The diagnostic value of serum IL-37 was further assessed through ROC curve analysis in the present study. The analysis identified an optimal cut-off point for serum IL-37 levels at 61.95 pg/ml, demonstrating maximum sensitivity and specificity (92.3% and 87.7% respectively) suggesting IL37 as a reliable biomarker for oral SCC screening. The ROC curve area of 0.90 further emphasizes IL-37's diagnostic accuracy in distinguishing oral SCC patients from healthy individuals. However, this is a preliminary study, and more studies with larger sample size is required to exactly detect the biological and the clinical roles of IL-37 in head and neck cancers, particularly oral SCC. 

## Conclusion

In summary, our study strengthens the evidence that IL-37 holds considerable potential as a tumor biomarker for oral SCC. Elevated IL-37 sera of patients with oral SCC highlight its role in the disease's pathology and its potential utility in early detection and screening. The study suggests that future research should investigate how IL-37 influences tumor behavior and examine its potential diagnostic/therapeutic applications.
